# Supplementary material for: Comparative effectiveness of alternative intervals between first and second doses of the mRNA COVID-19 vaccines
Source: Nat Commun. 2024 Feb 9;15:1214. doi: 10.1038/s41467-024-45334-8 (PMC10853518; doi:10.1038/s41467-024-45334-8)
Supplement: Supplementary file 1 — Supplementary Information [file 41467_2024_45334_MOESM1_ESM.pdf]

Supplementary Information

Comparative Effectiveness of Alternative Intervals between First and Second Doses of the mRNA COVID-19 Vaccines

AUTHOR LIST

Kayoko Shioda, PhD, DVM, MPH[1,2], Alexander Breskin, PhD, MPH[3,4], Pravara Harati[5], Allison Chamberlain, PhD, MS[6], Toshiaki Komura[7], Benjamin A Lopman, PhD[6], Elizabeth T. Rogawski McQuade, PhD, MSPH[6]

AFFILIATIONS

1. Department of Global Health, Boston University, Boston, MA, USA
2. Boston University Center on Emerging Infectious Diseases, Boston, MA, USA
3. Regeneron Pharmaceuticals, Tarrytown, NY, USA
4. Department of Epidemiology, University of North Carolina at Chapel Hill, Chapel Hill, NC, USA
5. Georgia Department of Public Health, Atlanta, GA, USA
6. Department of Epidemiology, Rollins School of Public Health, Emory University, Atlanta, GA, USA
7. Department of Epidemiology, Boston University, Boston, MA, USA

CORRESPONDING AUTHOR

Kayoko Shioda, PhD, DVM, MPH
Department of Global Health
Boston University School of Public Health
Boston University Center on Emerging Infectious Diseases (CEID)

SUPPLEMENTARY TABLES

Supplementary Table 1. Characteristics of the vaccine recipients stratified by interdose intervals in Georgia, United States, December 2020-March 2022 (N=6,128,364).

	Recommended (N=4,337,660)	Early (N= 38,539)	Allowable (N= 834,219)	Late (N= 140,348)	Late (No 2nd dose) (N= 717,051)	Overall (N=6,128,364)
**Sex**						
Female	2,360,779 (54.4%)	21,150 (54.9%)	449,392 (53.9%)	74,850 (53.3%)	355,804 (49.6%)	3,294,046 (53.8%)
Male	1,939,253 (44.7%)	17,185 (44.6%)	378,883 (45.4%)	64,574 (46.0%)	344,538 (48.0%)	2,772,105 (45.2%)
Unknown	37,628 (0.9%)	204 (0.5%)	5,944 (0.7%)	924 (0.7%)	16,709 (2.3%)	62,213 (1.0%)
**Race**						
White	2,185,474 (50.4%)	20,711 (53.7%)	419,817 (50.3%)	60,488 (43.1%)	319,285 (44.5%)	3,026,177 (49.4%)
Black	1,141,166 (26.3%)	9,515 (24.7%)	227,130 (27.2%)	48,470 (34.5%)	188,396 (26.3%)	1,636,871 (26.7%)
Asian	259,143 (6.0%)	1,896 (4.9%)	52,395 (6.3%)	5,277 (3.8%)	37,984 (5.3%)	360,254 (5.9%)
AIAN	15,021 (0.3%)	137 (0.4%)	3,307 (0.4%)	682 (0.5%)	3,037 (0.4%)	22,496 (0.4%)
NHPI	9,765 (0.2%)	72 (0.2%)	2,542 (0.3%)	243 (0.2%)	2,242 (0.3%)	15,020 (0.2%)
Other	590,168 (13.6%)	5,182 (13.4%)	104,788 (12.6%)	21,532 (15.3%)	117,319 (16.4%)	850,033 (13.9%)
Unknown	136,923 (3.2%)	1,026 (2.7%)	24,240 (2.9%)	3,656 (2.6%)	48,788 (6.8%)	217,513 (3.5%)
**Ethnicity**						
Hispanic	345,376 (8.0%)	2,129 (5.5%)	70,062 (8.4%)	15,272 (10.9%)	79,420 (11.1%)	521,415 (8.5%)
Non-Hispanic	3,722,921 (85.8%)	34,385 (89.2%)	713,014 (85.5%)	116,465 (83.0%)	528,351 (73.7%)	5,157,748 (84.2%)
Unknown	269,363 (6.2%)	2,025 (5.3%)	51,143 (6.1%)	8,611 (6.1%)	109,280 (15.2%)	449,201 (7.3%)
**Age (in years)**						
Mean (SD)	47.1 (20.7)	51.8 (19.6)	44.2 (20.5)	38.5 (19.6)	42.0 (20.1)	45.8 (20.7)
**Vaccine manufacturer**						
Moderna	1,744,606 (40.2%)	20,928 (54.3%)	256,562 (30.8%)	47,769 (34.0%)	250,511 (34.9%)	2,337,570 (38.1%)
Pfizer-BioNTech	2,593,054 (59.8%)	17,611 (45.7%)	577,657 (69.2%)	92,579 (66.0%)	466,540 (65.1%)	3,790,794 (61.9%)
**Prior infection**						
% with prior infection	8.4%	8.6%	9.1%	11.3%	6.8%	8.5%
**Public health district**						

01-1	187,072 (4.3%)	1,825 (4.7%)	44,184 (5.3%)	6,718 (4.8%)	21,331 (3.0%)	262,704 (4.3%)
01-2	169,069 (3.9%)	1,991 (5.2%)	31,852 (3.8%)	4,809 (3.4%)	16,380 (2.3%)	225,202 (3.7%)
02-0	254,191 (5.9%)	3,095 (8.0%)	45,260 (5.4%)	6,531 (4.7%)	23,437 (3.3%)	334,130 (5.5%)
03-1	368,195 (8.5%)	2,964 (7.7%)	97,357 (11.7%)	11,412 (8.1%)	39,994 (5.6%)	522,751 (8.5%)
03-2	454,674 (10.5%)	5,480 (14.2%)	88,560 (10.6%)	13,646 (9.7%)	50,959 (7.1%)	616,773 (10.1%)
03-3	97,041 (2.2%)	762 (2.0%)	18,638 (2.2%)	3,484 (2.5%)	12,108 (1.7%)	133,292 (2.2%)
03-4	468,484 (10.8%)	3,651 (9.5%)	95,626 (11.5%)	13,568 (9.7%)	47,461 (6.6%)	632,812 (10.3%)
03-5	321,575 (7.4%)	2,852 (7.4%)	68,106 (8.2%)	9,968 (7.1%)	37,935 (5.3%)	443,544 (7.2%)
04-0	292,338 (6.7%)	2,586 (6.7%)	53,445 (6.4%)	8,300 (5.9%)	30,160 (4.2%)	389,003 (6.3%)
05-1	39,986 (0.9%)	282 (0.7%)	10,431 (1.3%)	1,672 (1.2%)	4,927 (0.7%)	57,693 (0.9%)
05-2	185,768 (4.3%)	1,164 (3.0%)	32,220 (3.9%)	6,218 (4.4%)	21,358 (3.0%)	248,382 (4.1%)
06-0	165,848 (3.8%)	989 (2.6%)	30,573 (3.7%)	5,356 (3.8%)	18,263 (2.5%)	222,456 (3.6%)
07-0	118,027 (2.7%)	938 (2.4%)	19,828 (2.4%)	4,103 (2.9%)	15,010 (2.1%)	159,112 (2.6%)
08-1	73,324 (1.7%)	702 (1.8%)	9,547 (1.1%)	2,553 (1.8%)	8,060 (1.1%)	94,846 (1.5%)
08-2	125,448 (2.9%)	1,015 (2.6%)	20,849 (2.5%)	4,604 (3.3%)	13,086 (1.8%)	166,136 (2.7%)
09-1	231,647 (5.3%)	1,749 (4.5%)	30,606 (3.7%)	6,065 (4.3%)	24,408 (3.4%)	296,149 (4.8%)
09-2	100,049 (2.3%)	871 (2.3%)	16,032 (1.9%)	3,838 (2.7%)	11,751 (1.6%)	133,407 (2.2%)
10-0	180,854 (4.2%)	1,335 (3.5%)	29,871 (3.6%)	4,398 (3.1%)	15,561 (2.2%)	233,297 (3.8%)
Unknown	504,070 (11.6%)	4,288 (11.1%)	91,234 (10.9%)	23,105 (16.5%)	304,862 (42.5%)	956,675 (15.6%)
**Calendar month and year of the first dose administration**						
2020-12	101,949 (2.4%)	888 (2.3%)	5,793 (0.7%)	1,023 (0.7%)	2,225 (0.3%)	111,878 (1.8%)
2021-01	602,493 (13.9%)	8,151 (21.2%)	84,106 (10.1%)	6,957 (5.0%)	17,447 (2.4%)	719,154 (11.7%)
2021-02	387,145 (8.9%)	3,418 (8.9%)	43,446 (5.2%)	3,568 (2.5%)	15,332 (2.1%)	452,909 (7.4%)
2021-03	1,016,958 (23.4%)	9,281 (24.1%)	251,321 (30.1%)	12,163 (8.7%)	41,198 (5.7%)	1,330,921 (21.7%)
2021-04	693,456 (16.0%)	5,589 (14.5%)	136,574 (16.4%)	18,171 (12.9%)	64,925 (9.1%)	918,715 (15.0%)
2021-05	324,722 (7.5%)	2,104 (5.5%)	51,904 (6.2%)	14,744 (10.5%)	40,217 (5.6%)	433,691 (7.1%)
2021-06	167,568 (3.9%)	1,200 (3.1%)	31,862 (3.8%)	9,306 (6.6%)	27,599 (3.8%)	237,535 (3.9%)
2021-07	211,877 (4.9%)	1,485 (3.9%)	38,551 (4.6%)	11,539 (8.2%)	33,790 (4.7%)	297,242 (4.9%)

2021-08	341,297 (7.9%)	2,586 (6.7%)	69,870 (8.4%)	23,247 (16.6%)	81,032 (11.3%)	518,032 (8.5%)
2021-09	172,661 (4.0%)	1,379 (3.6%)	35,955 (4.3%)	14,064 (10.0%)	74,421 (10.4%)	298,480 (4.9%)
2021-10	68,634 (1.6%)	693 (1.8%)	15,066 (1.8%)	6,762 (4.8%)	57,893 (8.1%)	149,048 (2.4%)
2021-11	108,128 (2.5%)	616 (1.6%)	28,313 (3.4%)	8,797 (6.3%)	78,823 (11.0%)	224,677 (3.7%)
2021-12	63,172 (1.5%)	460 (1.2%)	23,942 (2.9%)	7,384 (5.3%)	109,593 (15.3%)	204,551 (3.3%)
2022-01	61,214 (1.4%)	487 (1.3%)	14,616 (1.8%)	2,623 (1.9%)	71,508 (10.0%)	152,804 (2.5%)
2022-02	16,386 (0.4%)	200 (0.5%)	2,900 (0.3%)	0 (0%)	1,048 (0.1%)	60,744 (1.0%)
2022-03	0 (0%)	2 (0.0%)	0 (0%)	0 (0%)	0 (0%)	17,983 (0.3%)

Abbreviations: AIAN, American Indian and Alaska Native Resources; NHIS, Native Hawaiian and Pacific Islander; SD, standard deviation.

Intervals between the 1st and 2nd doses: the “early” interval is ≤16 days for Pfizer-BioNTech and ≤23 days for Moderna; the “recommended” interval is17-25 days for Pfizer-BioNTech and 24-32 days for Moderna; the “late-but-allowable” interval is 26-42 days for Pfizer-BioNTech and 33-49 days for Moderna; the “late” interval is ≥43 days for Pfizer-BioNTech and ≥50 days for Moderna. The interval could not be determined for individuals who received the first dose close to the end of the study period, as enough time had not passed.

Definition of the public health district in Georgia:
01-1 Northwest (Rome)
01-2 North Georgia (Dalton)
02-0 North (Gainesville)
03-1 Cobb-Douglas
03-2 Fulton
03-3 Clayton (Jonesboro)
03-4 GNR (Lawrenceville)
03-5 DeKalb
04-0 District 4
05-1 South Central (Dublin)
05-2 North Central (Macon)
06-0 East Central (Augusta)
07-0 West Central (Columbus)
08-1 South (Valdosta)
08-2 Southwest (Albany)
09-1 Coastal (Savannah)
09-2 Southeast (Waycross)
10-0 Northeast (Athens)

Supplementary Table 2. Timing of SARS-CoV-2 infection relative to vaccination by intervals between the first and second doses of mRNA COVID-19 vaccines.

	Recommended	Early	Late-but-allowable	Late	Late (No 2nd dose)	Overall
Timing of infection	(N=4,337,660)	(N= 38,539)	(N= 834,219)	(N= 140,348)	(N= 717,051)	(N=6,128,364)
After 2nd dose	316,635 (7.3%)	3,053 (7.9%)	60,848 (7.3%)	7,583 (5.4%)	0 (0%)	388,119 (6.3%)
Between 1st and 2nd	10,581 (0.2%)	59 (0.2%)	6,822 (0.8%)	8,793 (6.3%)	0 (0%)	26,255 (0.4%)
Before 1st dose	363,057 (8.4%)	3,298 (8.6%)	76,151 (9.1%)	15,853 (11.3%)	49,082 (6.8%)	517,966 (8.5%)
no infection	4,010,444 (92.5%)	35,427 (91.9%)	766,549 (91.9%)	123,972 (88.3%)	678,962 (94.7%)	5,675,827 (92.6%)
Missing	0 (0%)	0 (0%)	0 (0%)	0 (0%)	38,089 (5.3%)	38,163 (0.6%)

NOTE: The percentages in each column do not add up to 100% because people could be infected before vaccination and also after vaccination.

Intervals between the 1st and 2nd doses: the “early” interval is ≤16 days for Pfizer-BioNTech and ≤23 days for Moderna; the “recommended” interval is17-25 days for Pfizer-BioNTech and 24-32 days for Moderna; the “late-but-allowable” interval is 26-42 days for Pfizer-BioNTech and 33-49 days for Moderna; the “late” interval is ≥43 days for Pfizer-BioNTech and ≥50 days for Moderna.

Supplementary Table 3. mRNA COVID-19 vaccines interdose intervals by race.

	White	Black	Asian	AIAN	NHPI	Other	Unknown	Overall
	(N=3,026,177)	(N=1,636,871)	(N= 360,254)	(N= 22,496)	(N= 15,020)	(N= 850,033)	(N= 217,513)	(N=6,128,364)
**Interval between the 1st and 2nd doses**								
Recommended	2,185,474 (72.2%)	1,141,166 (69.7%)	259,143 (71.9%)	15,021 (66.8%)	9,765 (65.0%)	590,168 (69.4%)	136,923 (62.9%)	4,337,660 (70.8%)
Early	20,711 (0.7%)	9,515 (0.6%)	1,896 (0.5%)	137 (0.6%)	72 (0.5%)	5,182 (0.6%)	1,026 (0.5%)	38,539 (0.6%)
Late-but-allowable	419,817 (13.9%)	227,130 (13.9%)	52,395 (14.5%)	3,307 (14.7%)	2,542 (16.9%)	104,788 (12.3%)	24,240 (11.1%)	834,219 (13.6%)
Late	379,773 (12.5%)	236,866 (14.5%)	43,261 (12.0%)	3,719 (16.5%)	2,485 (16.5%)	138,851 (16.3%)	52,444 (24.1%)	857,399 (14.0%)
Missing	20402 (0.7%)	22194 (1.4%)	3559 (1.0%)	312 (1.4%)	156 (1.0%)	11044 (1.3%)	2880 (1.3%)	60547 (1.0%)

Abbreviations: AIAN, American Indian and Alaska Native Resources; NHIS, Native Hawaiian and Pacific Islander.

Intervals between the 1st and 2nd doses: the “early” interval is ≤16 days for Pfizer-BioNTech and ≤23 days for Moderna; the “recommended” interval is17-25 days for Pfizer-BioNTech and 24-32 days for Moderna; the “late-but-allowable” interval is 26-42 days for Pfizer-BioNTech and 33-49 days for Moderna; the “late” interval is ≥43 days for Pfizer-BioNTech and ≥50 days for Moderna.

Supplementary Table 4. Definition of protocols based on the intervals between the 1st and 2nd dose.

	Main analysis		Sensitivity analysis
	**Moderna**	**Pfizer-BioNTech**	**Pfizer-BioNTech**
Recommended	24-32 days	17-25 days	24-32 days
Late-but-allowable	33-49 days	26-42 days	33-49 days
Late	≥50 days	≥43 days	≥50 days

Supplementary Table 5. Condition on censoring in each of three copies of the longitudinal dataset corresponding to the mRNA COVID-19 vaccine protocols

**Protocol-specific copy**	**Pfizer-BioNTech**	**Moderna**
Recommended	1) For those who received their 2nd dose between Day 17-25 from the receipt of their 1st dose, they were censored at the end of study period unless they had COVID-19 by then<br><br>2) For those who received their 2nd dose on Day 16 or before from the receipt of their 1st dose, they were censored on the day of the receipt of their 2nd dose unless they had COVID-19 by then<br><br>3) For those who received their 2nd dose on Day 26 or after from the receipt of their 1st dose, they were censored on Day 25 from the receipt of their first dose unless they had COVID-19 by then<br><br>4) For those who had not received their 2nd dose by the end of the study period and if the number of days from the date of their 1st dose administration to the end of study period is <26 days, they were censored on the last day of the study period unless they had COVID-19 by then<br><br>5) For those who had not received their 2nd dose by the end of the study period and if the number of days from the date of their 1st dose administration to the end of study period is ≥26 days, they were censored on Day 25 from the receipt of their 1st dose unless they had COVID-19 by then	1) For those who received their 2nd dose between Day 24-32 from the receipt of their 1st dose, they were censored at the end of study period unless they had COVID-19 by then<br><br>2) For those who received their 2nd dose on Day 23 or before from the receipt of their 1st dose, they were censored on the day of the receipt of their 2nd dose unless they had COVID-19 by then<br><br>3) For those who received their 2nd dose on Day 33 or after from the receipt of their 1st dose, they were censored on Day 32 from the receipt of their first dose unless they had COVID-19 by then<br><br>4) For those who had not received their 2nd dose by the end of the study period and if the number of days from the date of their 1st dose administration to the end of study period is <33 days, they were censored on the last day of the study period unless they had COVID-19 by then<br><br>5) For those who had not received their 2nd dose by the end of the study period and if the number of days from the date of their 1st dose administration to the end of study period is ≥33 days, they were censored on Day 32 from the receipt of their 1st dose unless they had COVID-19 by then
Late but allowable	1) For those who received their 2nd dose between Day 26-42 from the receipt of their 1st dose, they were censored at the end of study period	1) For those who received their 2nd dose between Day 33-49 from the receipt of their 1st dose, they were censored at the end of study period

	unless they had COVID-19 by then<br><br>2) For those who received their 2nd dose on Day 25 or before from the receipt of their 1st dose, they were censored on the day of the receipt of their 2nd dose unless they had COVID-19 by then<br><br>3) For those who received their 2nd dose on Day 43 or after from the receipt of their 1st dose, they were censored on Day 42 from the receipt of their first dose unless they had COVID-19 by then<br><br>4) For those who had not received their 2nd dose by the end of the study period and if the number of days from the date of their 1st dose administration to the end of study period is <43 days, they were censored on the last day of the study period unless they had COVID-19 by then<br><br>5) For those who had not received their 2nd dose by the end of the study period and if the numbernumer of days from the date of their 1st dose administration to the end of study period is ≥43 days, they were censored on Day 42 from the receipt of their 1st dose unless they had COVID-19 by then	unless they had COVID-19 by then<br><br>2) For those who received their 2nd dose on Day 32 or before from the receipt of their 1st dose, they were censored on the day of the receipt of their 2nd dose unless they had COVID-19 by then<br><br>3) For those who received their 2nd dose on Day 50 or after from the receipt of their 1st dose, they were censored on Day 49 from the receipt of their first dose unless they had COVID-19 by then<br><br>4) For those who had not received their 2nd dose by the end of the study period and if the number of days from the date of their 1st dose administration to the end of study period is <50 days, they were censored on the last day of the study period unless they had COVID-19 by then<br><br>5) For those who had not received their 2nd dose by the end of the study period and if the numbernumer of days from the date of their 1st dose administration to the end of study period is ≥50 days, they were censored on Day 49 from the receipt of their 1st dose unless they had COVID-19 by then
Late	1) For those who received their 2nd dose between Day 43 or after from the receipt of their 1st dose, they were censored at the end of study period unless they had COVID-19 by then<br><br>2) For those who received their 2nd dose on Day 42 or before from the receipt of their 1st dose, they were censored on the day of the receipt of their 2nd dose unless they had COVID-19 by then	1) For those who received their 2nd dose between Day 50 or after from the receipt of their 1st dose, they were censored at the end of study period unless they had COVID-19 by then<br><br>2) For those who received their 2nd dose on Day 49 or before from the receipt of their 1st dose, they were censored on the day of the receipt of their 2nd dose unless they had COVID-19 by then

	3) For those who had not received their 2nd dose by the end of the study period and if the numbernumer of days from the date of their 1st dose administration to the end of study period is ≥43 days, they were censored on the last day of the study period unless they had COVID-19 by then	3) For those who had not received their 2nd dose by the end of the study period and if the numbernumer of days from the date of their 1st dose administration to the end of study period is ≥50 days, they were censored on the last day of the study period unless they had COVID-19 by then

SUPPLEMENTARY FIGURES

Supplementary Figure 1. Estimates of inverse probability of censoring-weighted cumulative risk functions of SARS-CoV-2 infection by protocol for Pfizer-BioNTech and Moderna, stratified by the presence of reported prior infection.

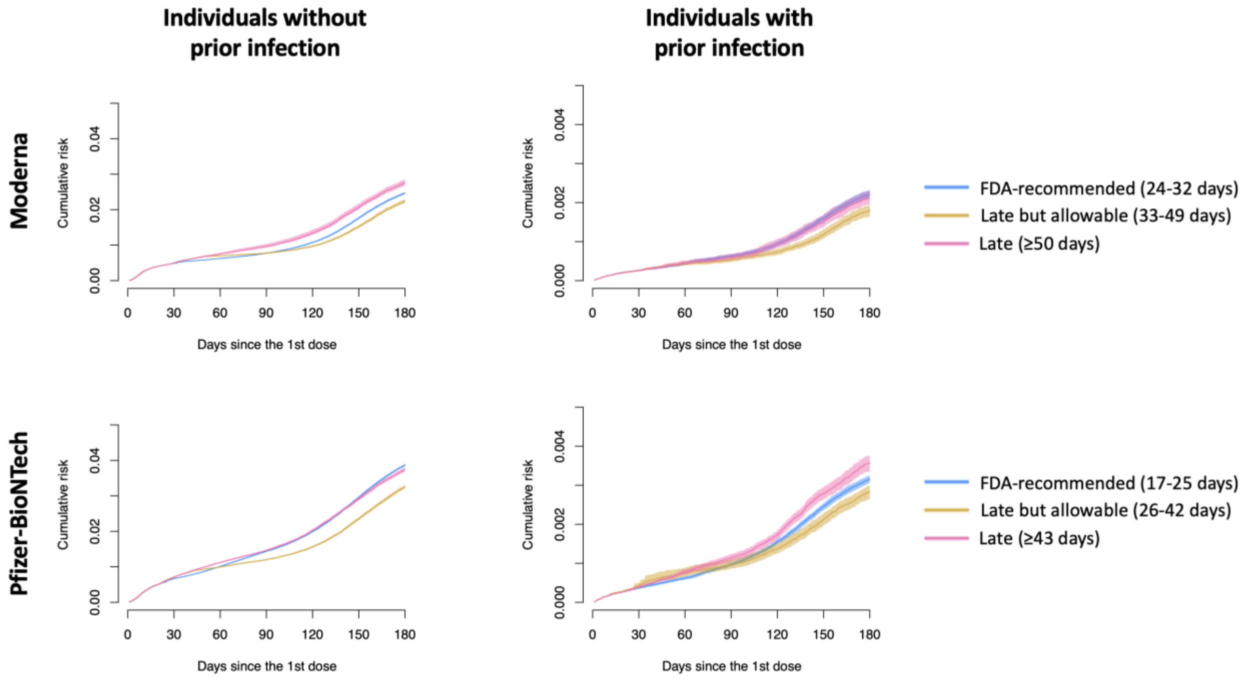

Data are presented as point estimates (solid lines) and 95% confidence intervals (shaded areas represent) using a nonparametric bootstrap based on 200 resamples.

*Please note that the scale for Y axes in the panels for the individuals with prior infection is different from that for individuals without prior infection.

Supplementary Figure 2. Estimates of inverse probability of censoring-weighted cumulative risk functions of SARS-CoV-2 infection by protocol for Pfizer-BioNTech and Moderna, stratified by age group.

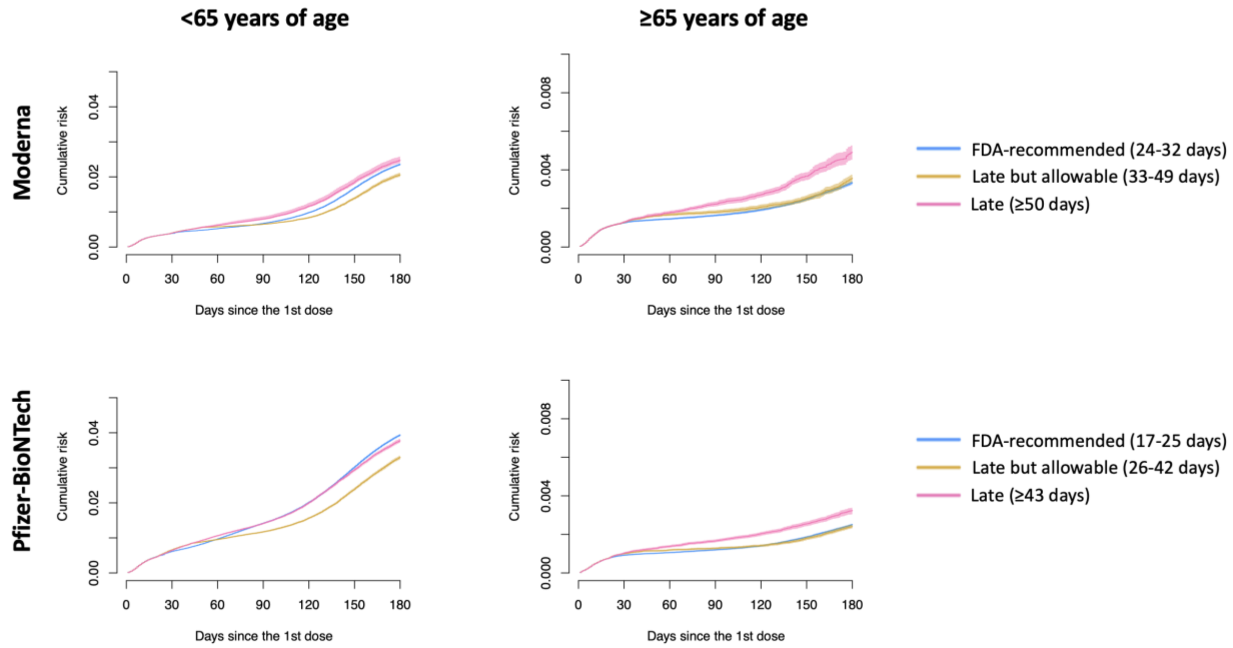

Data are presented as point estimates (solid lines) and 95% confidence intervals (shaded areas represent) using a nonparametric bootstrap based on 200 resamples.

*Please note that the scale for Y axes in the panels is different in each age group.

Supplementary Figure 3. Results of the sensitivity analysis (creating the "first dose only" protocol): Estimates of inverse probability of censoring-weighted cumulative risk functions of SARS-CoV-2 infection by protocol for Pfizer-BioNTech (a) and Moderna (b).

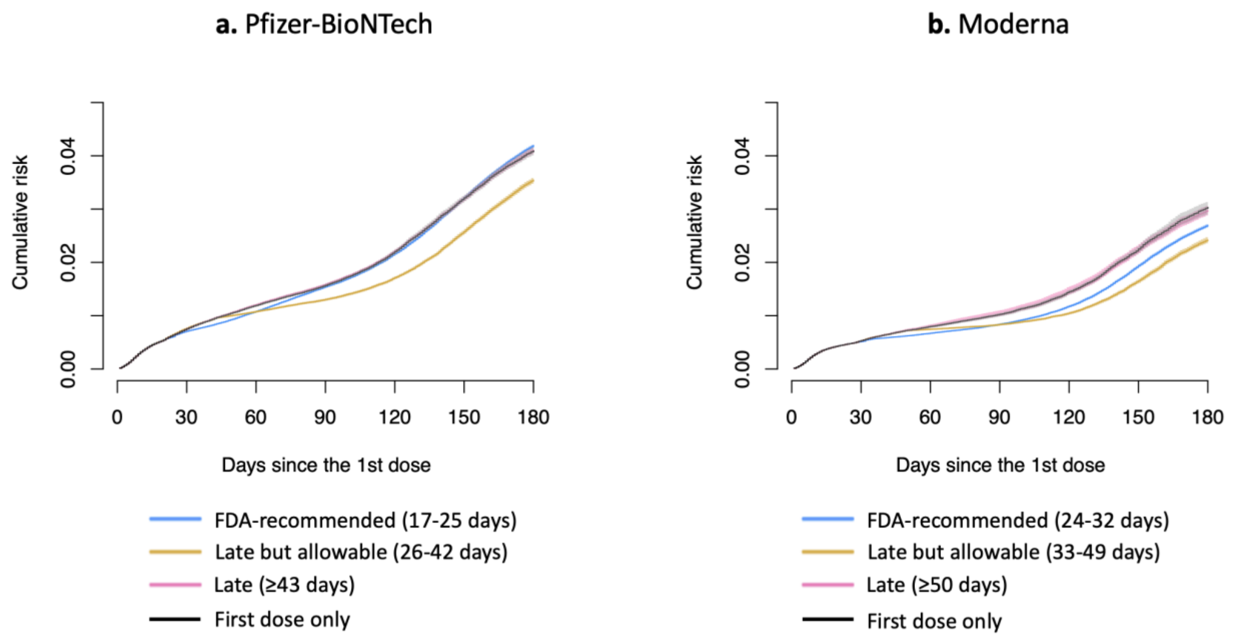

Data are presented as point estimates (solid lines) and 95% confidence intervals (shaded areas represent) using a nonparametric bootstrap based on 200 resamples.

Supplementary Figure 4. Results of the sensitivity analysis (comparative effectiveness up to November 2021 (pre-Omicron)): Estimates of inverse probability of censoring-weighted cumulative risk functions of SARS-CoV-2 infection by protocol for Pfizer-BioNTech (a) and Moderna (b).

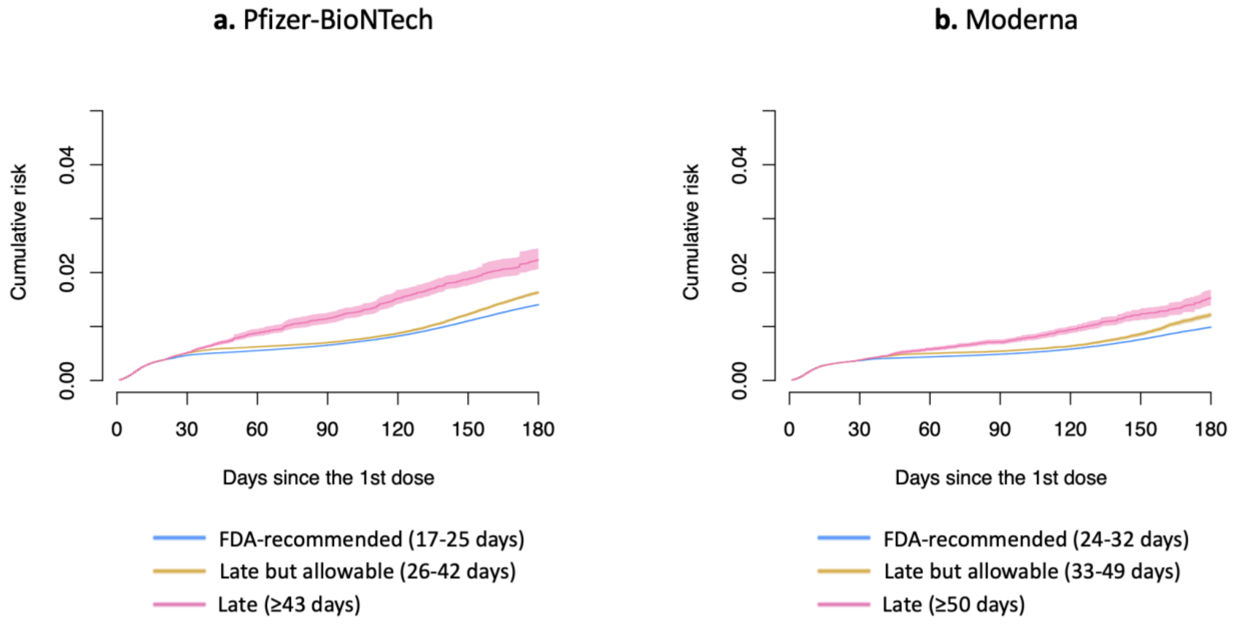

Data are presented as point estimates (solid lines) and 95% confidence intervals (shaded areas represent) using a nonparametric bootstrap based on 200 resamples.

Supplementary Figure 5. Results of the sensitivity analysis (comparative effectiveness up to September 2021 (before the booster dose became available)): Estimates of inverse probability of censoring-weighted cumulative risk functions of SARS-CoV-2 infection by protocol for Pfizer-BioNTech (a) and Moderna (b).

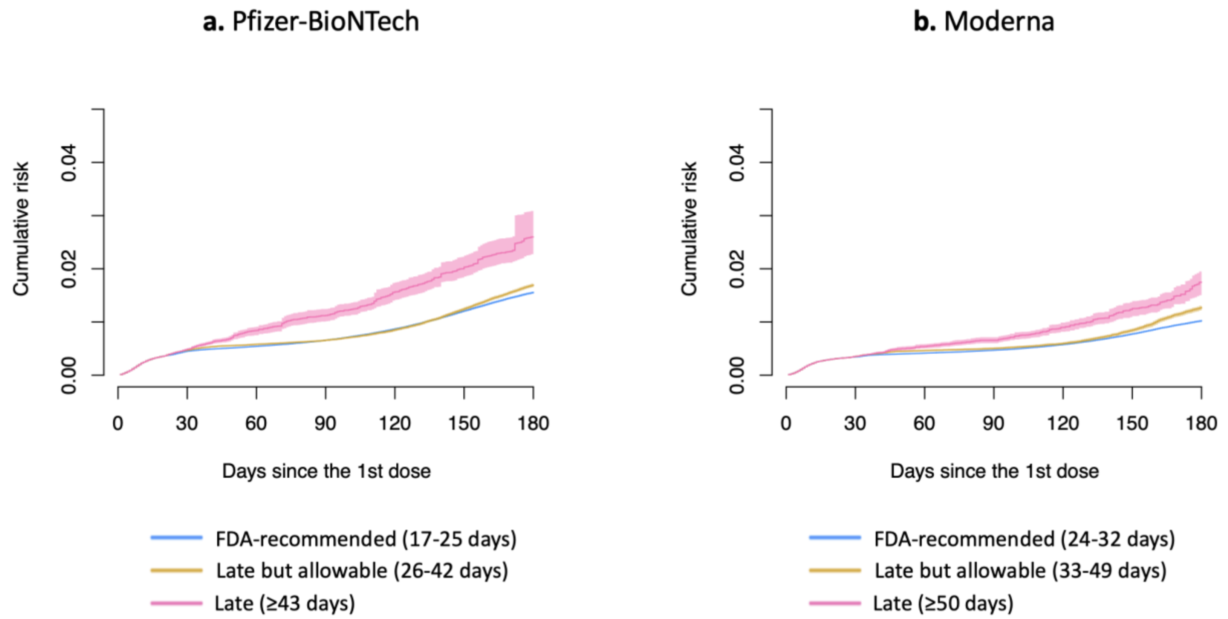

Data are presented as point estimates (solid lines) and 95% confidence intervals (shaded areas represent) using a nonparametric bootstrap based on 200 resamples.

Supplementary Figure 6. Results of the sensitivity analysis (ending the follow-up period at the earliest of SARS-CoV-2 infection, protocol nonadherence, or 180 days after the first dose administration): Estimates of inverse probability of censoring-weighted cumulative risk functions of SARS-CoV-2 infection by protocol for Pfizer-BioNTech (a) and Moderna (b).

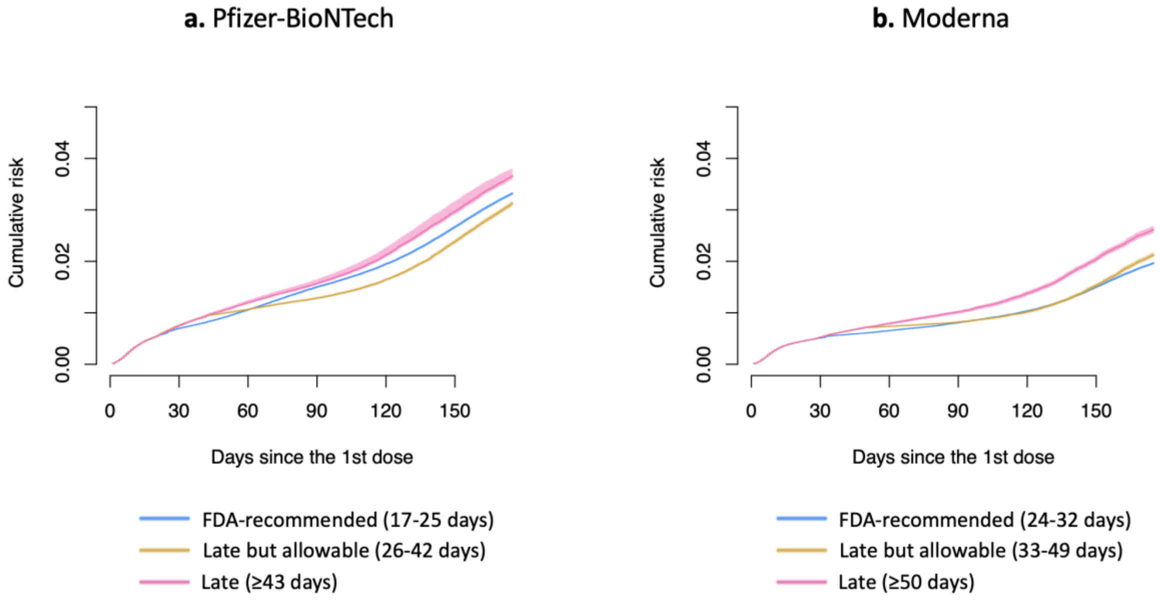

Data are presented as point estimates (solid lines) and 95% confidence intervals (shaded areas represent) using a nonparametric bootstrap based on 200 resamples.

Supplementary Figure 7. Results of the sensitivity analysis (excluding individuals with unknown characteristics): Estimates of inverse probability of censoring-weighted cumulative risk functions of SARS-CoV-2 infection by protocol for Pfizer-BioNTech (a) and Moderna (b).

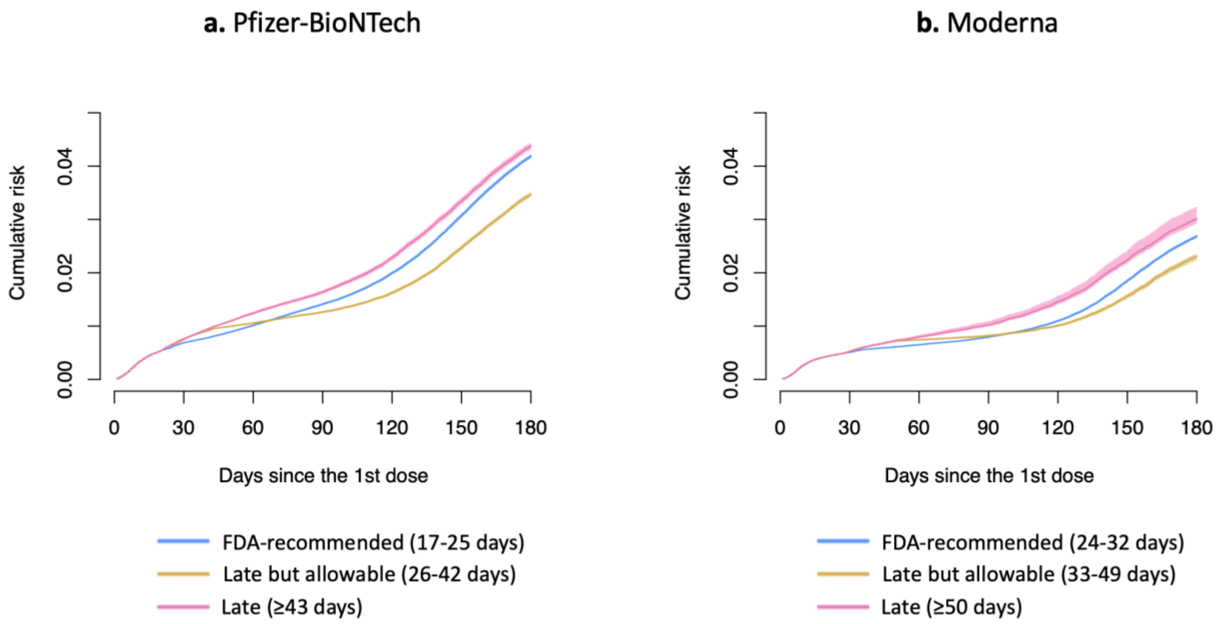

Data are presented as point estimates (solid lines) and 95% confidence intervals (shaded areas represent) using a nonparametric bootstrap based on 200 resamples.

Supplementary Figure 8. Results of the sensitivity analysis (without excluding people who received their second dose >180 days after their first dose administration): Estimates of inverse probability of censoring-weighted cumulative risk functions of SARS-CoV-2 infection by protocol for Pfizer-BioNTech (a) and Moderna (b).

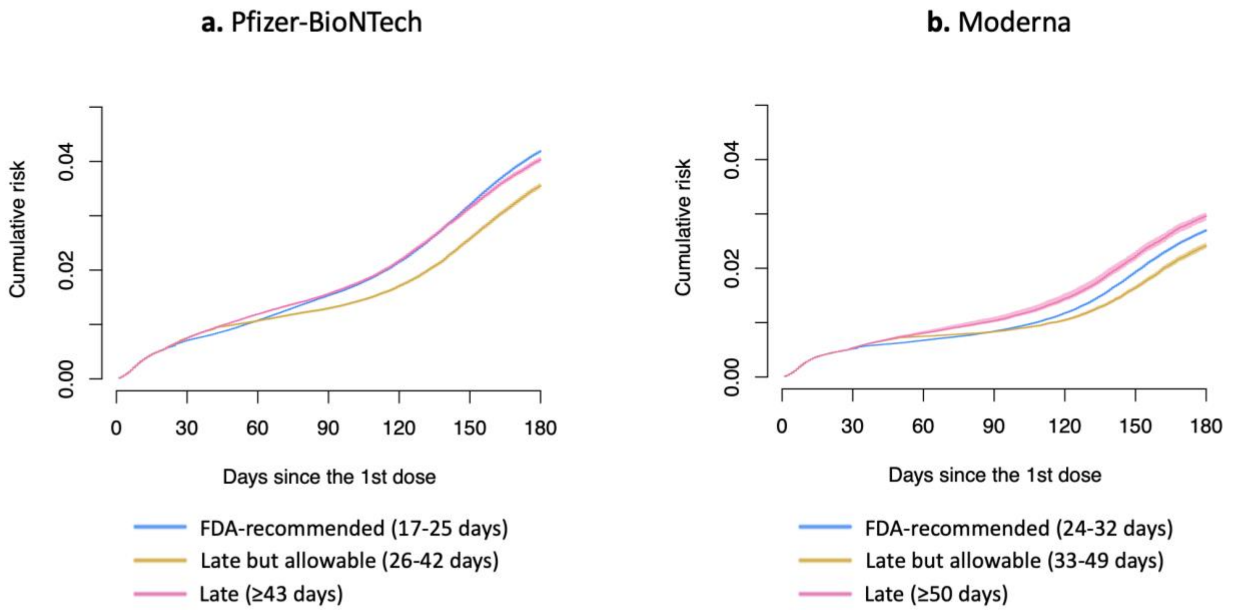

Data are presented as point estimates (solid lines) and 95% confidence intervals (shaded areas represent) using a nonparametric bootstrap based on 200 resamples.

Supplementary Figure 9. Results of the sensitivity analysis (natural splines for age and the date of the first dose administration to calculate the probability of being censored): Estimates of inverse probability of censoring-weighted cumulative risk functions of SARS-CoV-2 infection by protocol for Pfizer-BioNTech (a) and Moderna (b).

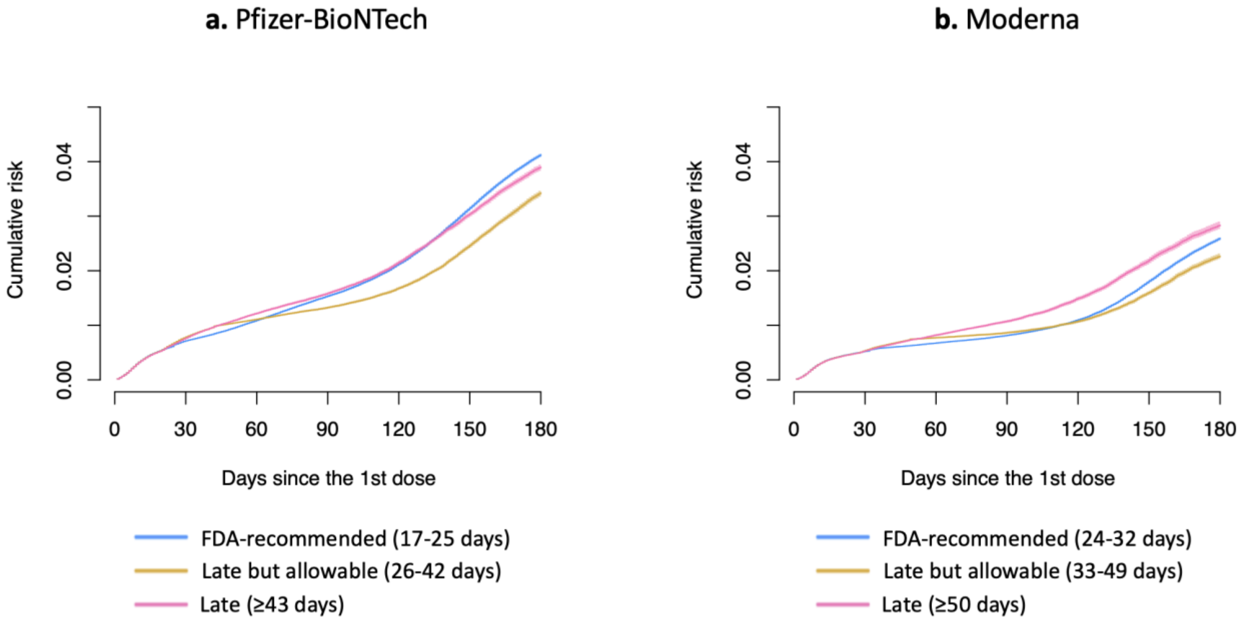

Data are presented as point estimates (solid lines) and 95% confidence intervals (shaded areas represent) using a nonparametric bootstrap based on 200 resamples.

Supplementary Figure 10. Results of the sensitivity analysis (1000 bootstrap iterations): Estimates of inverse probability of censoring-weighted cumulative risk functions of SARS-CoV-2 infection by protocol for Pfizer-BioNTech (a) and Moderna (b).

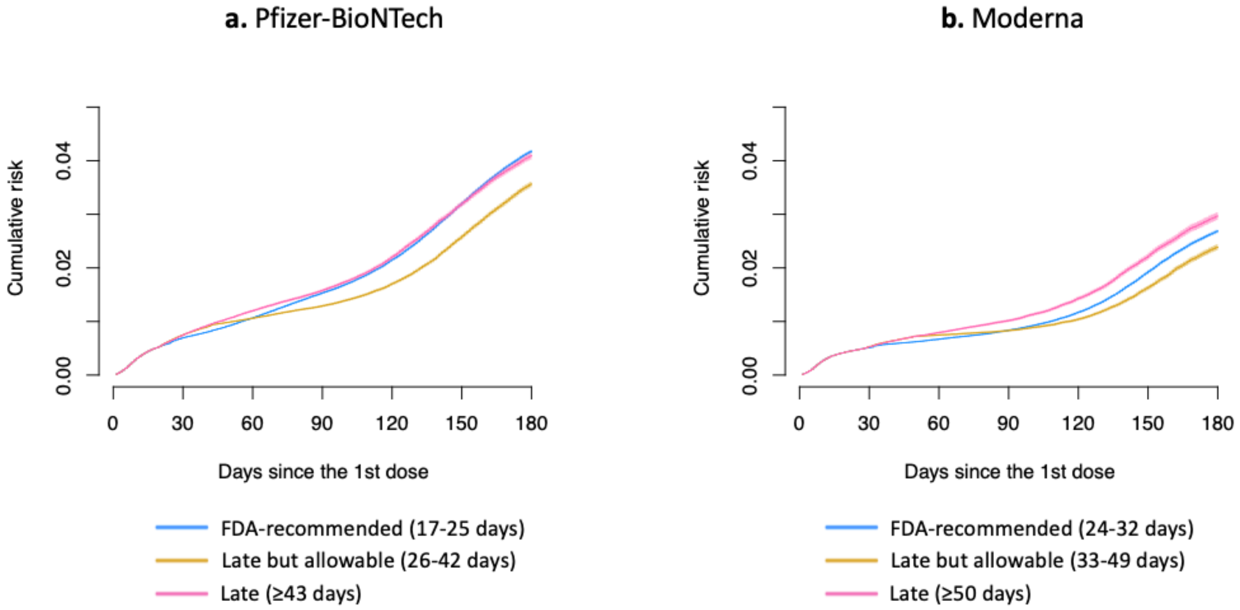

Data are presented as point estimates (solid lines) and 95% confidence intervals (shaded areas represent) using a nonparametric bootstrap based on 1000 resamples.

Supplementary Figure 11. Date of the second dose administration for people who received the 2nd dose >180 days after their 1st dose administration.

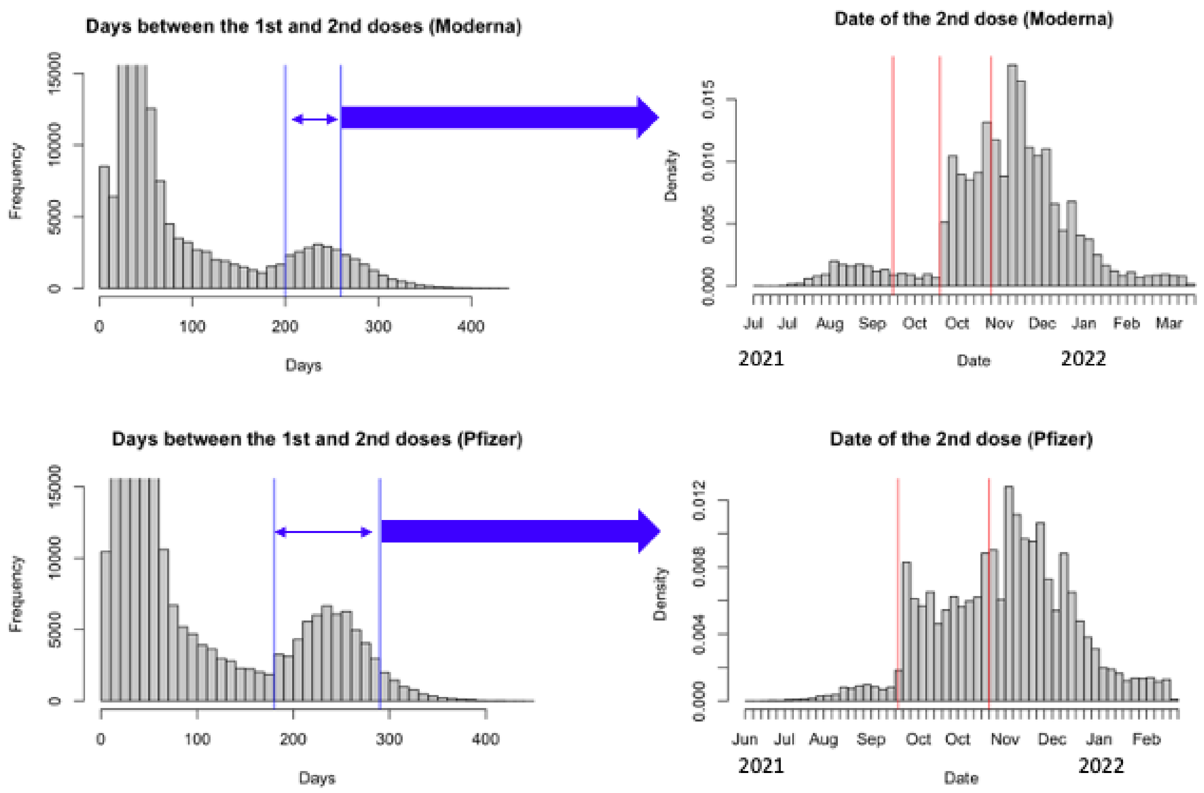

Red vertical lines:
2021-09-22: PFR booster available for high-risk groups
2021-10-20: MOD booster available for high-risk groups
2021-11-19: Booster available for general public
