# Supplementary material for: Comparative effectiveness of alternative intervals between first and second doses of the mRNA COVID-19 vaccines
Source: Nat Commun. 2024 Feb 9;15:1214. doi: 10.1038/s41467-024-45334-8 (PMC10853518; doi:10.1038/s41467-024-45334-8)
Supplement: Supplementary file 3 — Reporting Summary [file 41467_2024_45334_MOESM3_ESM.pdf]

nature portfolio

Corresponding author(s): Kayoko Shioda

Last updated by author(s): Jan 11, 2024

Reporting Summary

Nature Portfolio wishes to improve the reproducibility of the work that we publish. This form provides structure for consistency and transparency in reporting. For further information on Nature Portfolio policies, see our Editorial Policies and the Editorial Policy Checklist.

Statistics

For all statistical analyses, confirm that the following items are present in the figure legend, table legend, main text, or Methods section.

n/a	Confirmed	
☐	☒	The exact sample size (*n*) for each experimental group/condition, given as a discrete number and unit of measurement
☐	☒	A statement on whether measurements were taken from distinct samples or whether the same sample was measured repeatedly
☒	☐	The statistical test(s) used AND whether they are one- or two-sided<br>*Only common tests should be described solely by name; describe more complex techniques in the Methods section.*
☐	☒	A description of all covariates tested
☐	☒	A description of any assumptions or corrections, such as tests of normality and adjustment for multiple comparisons
☐	☒	A full description of the statistical parameters including central tendency (e.g. means) or other basic estimates (e.g. regression coefficient) AND variation (e.g. standard deviation) or associated estimates of uncertainty (e.g. confidence intervals)
☒	☐	For null hypothesis testing, the test statistic (e.g. *F*, *t*, *r*) with confidence intervals, effect sizes, degrees of freedom and *P* value noted<br>*Give P values as exact values whenever suitable.*
☒	☐	For Bayesian analysis, information on the choice of priors and Markov chain Monte Carlo settings
☐	☒	For hierarchical and complex designs, identification of the appropriate level for tests and full reporting of outcomes
☒	☐	Estimates of effect sizes (e.g. Cohen's *d*, Pearson's *r*), indicating how they were calculated

Our web collection on statistics for biologists contains articles on many of the points above.

Software and code

Policy information about availability of computer code

Data collection	No software was used for data collection
Data analysis	All analyses were conducted with R (R Center for Statistical Computing; Vienna, Austria) v4.2.1. The survival analysis was conducted using the 'survival' package as cited in the main text. R scripts can be found at the following github repository: https://github.com/KayokoShioda/COVID_mRNA_TTE_2ndDose.

For manuscripts utilizing custom algorithms or software that are central to the research but not yet described in published literature, software must be made available to editors and reviewers. We strongly encourage code deposition in a community repository (e.g. GitHub). See the Nature Portfolio guidelines for submitting code & software for further information.

Data

Policy information about availability of data

All manuscripts must include a data availability statement. This statement should provide the following information, where applicable:

- Accession codes, unique identifiers, or web links for publicly available datasets
- A description of any restrictions on data availability
- For clinical datasets or third party data, please ensure that the statement adheres to our policy

Individual-level data on COVID-19 test results from the Georgia State Electronic Notifiable Disease Surveillance System (SendSS) and COVID-19 vaccination from the

Georgia Department of Public Health (GDPH) are also not publicly available for privacy and ethical and legal issues, but aggregated data are available on the GDPH COVID-19 website (https://dph.georgia.gov/covid-19-status-report).

Research involving human participants, their data, or biological material

Policy information about studies with human participants or human data. See also policy information about sex, gender (identity/presentation), and sexual orientation and race, ethnicity and racism.

Reporting on sex and gender	There were 6,128,364 recipients of mRNA COVID-19 vaccines in Georgia who were included in our analysis, and 3,294,046 (53.8%) of them were female (Table 2).
Reporting on race, ethnicity, or other socially relevant groupings	There were 6,128,364 recipients of mRNA COVID-19 vaccines in Georgia who were included in our analysis, and 3,026,177 (49.4%) were White, 1,636,871 (26.7%) were Black, 360,254 (5.9%) were Asian, 22,496 (0.4%) were AIAN, 15,020 (0.2%) were NHPI, 850,033 (13.9%) were Other, and 217,513 (3.5%) were unknown, as shown in Table 2.
Population characteristics	Our study population included individuals who received at least one dose of an mRNA COVID-19 vaccine between December 13, 2020 and March 16, 2022 in Georgia, US. Population characteristics, such as age, sex, race, ethnicity, etc. can be found in Table 2.
Recruitment	Not applicable, as this was the surveillance data and vaccine registry data.
Ethics oversight	This activity was determined by the Georgia Department of Public Health (GDPH) Institutional Review Board to be non-research and consistent with public health surveillance as per title 45 code of Federal Regulations 46.102(l)(2).

Note that full information on the approval of the study protocol must also be provided in the manuscript.

Field-specific reporting

Please select the one below that is the best fit for your research. If you are not sure, read the appropriate sections before making your selection.

☒ Life sciences ☐ Behavioural & social sciences ☐ Ecological, evolutionary & environmental sciences

For a reference copy of the document with all sections, see nature.com/documents/nr-reporting-summary-flat.pdf

Life sciences study design

All studies must disclose on these points even when the disclosure is negative.

Sample size	Sample size calculation was not performed. We included all mRNA vaccine recipients in GA that meet our criteria (i.e., who received at least one dose of mRNA vaccine by March 16, 2022).
Data exclusions	Unvaccinated people were excluded because our goal was to evaluate vaccine effectiveness depending on different interdose intervals. We excluded 4,374 (0.1% of mRNA COVID-19 vaccine recipients) people who received their second dose ≤3 days after their first dose because of likely data entry errors. We also excluded 89,885 (1.4%) individuals who received their second dose more than 180 days after their first dose since an interdose interval of that length or longer is unlikely to be recommended and because individuals who received their second dose beyond this time likely received a booster dose at that time while their true second dose was received outside of Georgia or otherwise misrecorded (eFigure 1). Children <5 years of age were excluded as they were not eligible for COVID-19 vaccination during our study period and their primary dosing schedule was different from those for people ≥5 years of age. Recipients of non-mRNA COVID-19 vaccines (e.g., Janssen (Johnson & Johnson) vaccine and Novavax vaccine) were not included in the study.
Replication	We conducted more than 10 different sensitivity analyses and confirmed that results are robust, as stated in the main text.
Randomization	We employed a trial emulation approach (clone-censor-weight analysis) to understand how the different intervals between the first and second doses of the primary series of mRNA COVID-19 vaccines may change the risk of SARS-CoV-2 infection after the first dose administration. We created three copies of the longitudinal dataset corresponding to the three mRNA COVID-19 vaccination protocols of interest (FDA-recommended, late but allowable, and late). This method addresses measured confounding at baseline because the copies of each observation are identical at the start of follow-up. In each protocol-specific copy, a vaccine recipient who did not follow a given protocol was considered nonadherent and was censored at the time their vaccination course differed from the protocol.
Blinding	Blinding was not performed as it is a retrospective study using a trial emulation approach.

Reporting for specific materials, systems and methods

We require information from authors about some types of materials, experimental systems and methods used in many studies. Here, indicate whether each material, system or method listed is relevant to your study. If you are not sure if a list item applies to your research, read the appropriate section before selecting a response.

Materials & experimental systems

n/a	Involved in the study
☒	☐ Antibodies
☒	☐ Eukaryotic cell lines
☒	☐ Palaeontology and archaeology
☒	☐ Animals and other organisms
☐	☒ Clinical data
☒	☐ Dual use research of concern
☒	☐ Plants

Methods

n/a	Involved in the study
☒	☐ ChIP-seq
☒	☐ Flow cytometry
☒	☐ MRI-based neuroimaging

Clinical data

Policy information about clinical studies

All manuscripts should comply with the ICMJE guidelines for publication of clinical research and a completed CONSORT checklist must be included with all submissions.

Clinical trial registration	Not applicable as it is not a clinical trial.
Study protocol	Not applicable as it is not a clinical trial. The protocol of our trial emulation method can be found in the main text and supplement.
Data collection	Data were collected in Georgia, USA, from December 2020 to March 2022
Outcomes	Our outcome was SARS-CoV-2 infection defined as a positive result of real-time reverse transcriptase PCR test or antigen test. For the trial emulation approach, the follow-up period began the day after the index date (i.e., the day each individual received their first dose of mRNA COVID-19 vaccine) and ended at the earliest of SARS-CoV-2 infection, protocol nonadherence, or end of study (March 16, 2022). For the Cox PH model, the index date was the date of the second dose administration and ended at the earliest of SARS-CoV-2 infection or end of study (March 16, 2022).

Plants

Seed stocks	Not used.
Novel plant genotypes	Not applicable
Authentication	Not applicable
